# Supplementary material for: Soluble Guanylate Cyclase β1 Subunit Represses Human Glioblastoma Growth
Source: Cancers (Basel). 2023 Mar 2;15(5):1567. doi: 10.3390/cancers15051567 (PMC10001022; doi:10.3390/cancers15051567)
Supplement: Supplementary file 1 [file cancers-15-01567-s001.zip › cancers-2104792-Supplementary File S1.pptx]

## Slide 1
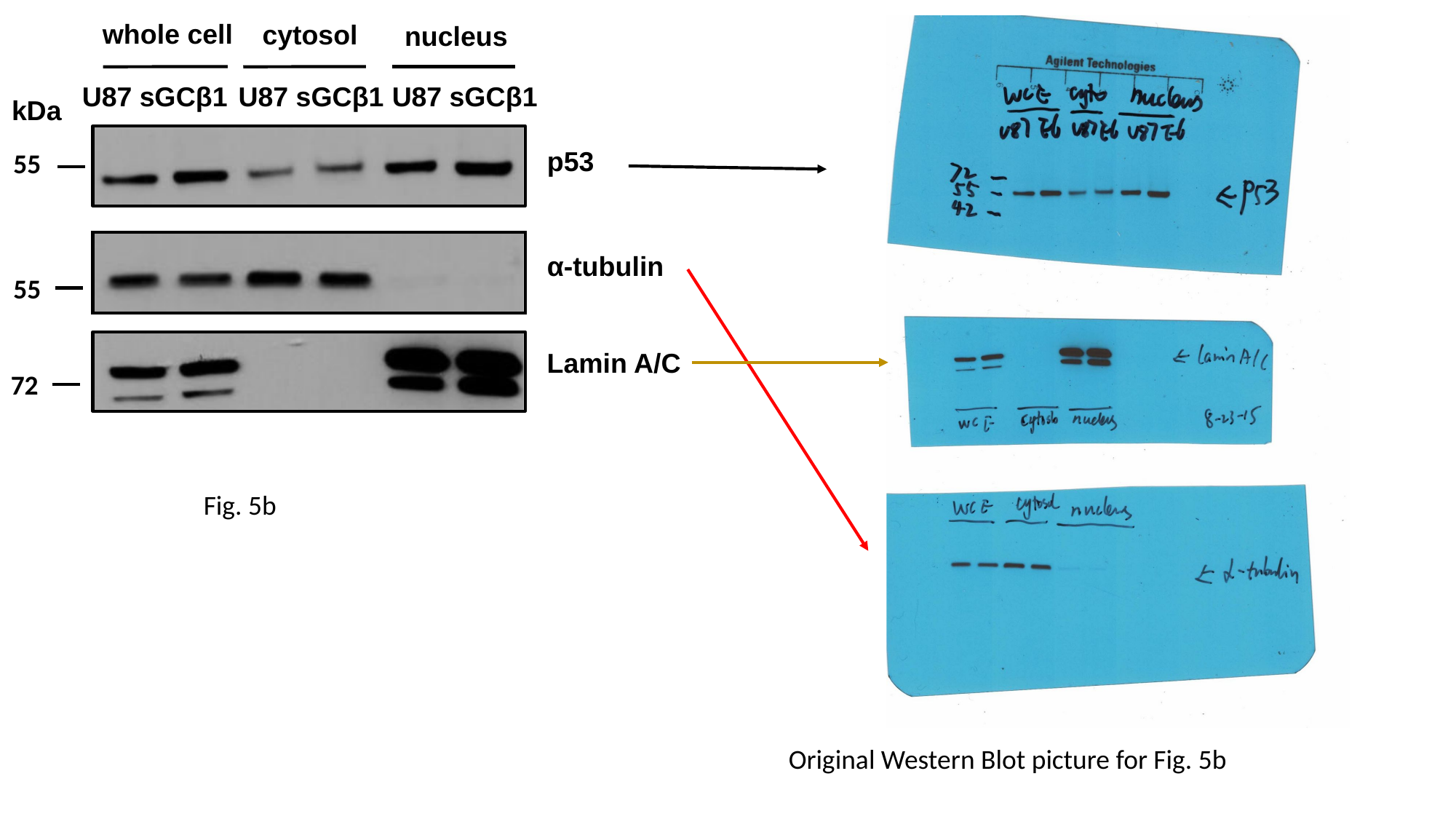

whole cell
cytosol
nucleus
U87 sGCβ1
U87 sGCβ1
U87 sGCβ1
kDa
p53
55
α-tubulin
55
Lamin A/C
72
Fig. 5b
Original Western Blot picture for Fig. 5b

## Slide 2
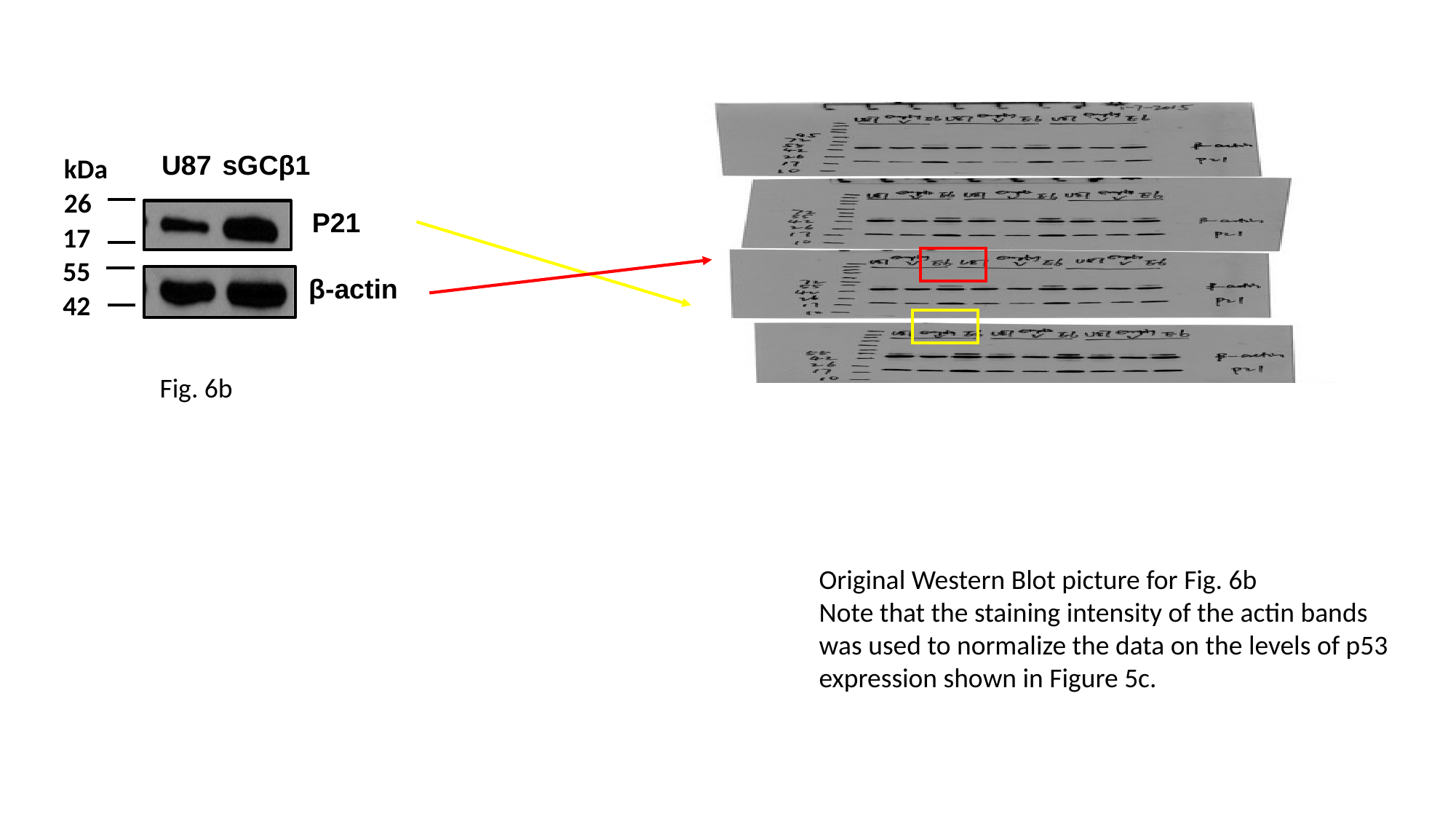

U87
sGCβ1
kDa
26
P21
17
55
β-actin
42
Fig. 6b
Original Western Blot picture for Fig. 6b
Note that the staining intensity of the actin bands was used to normalize the data on the levels of p53 expression shown in Figure 5c.

## Slide 3
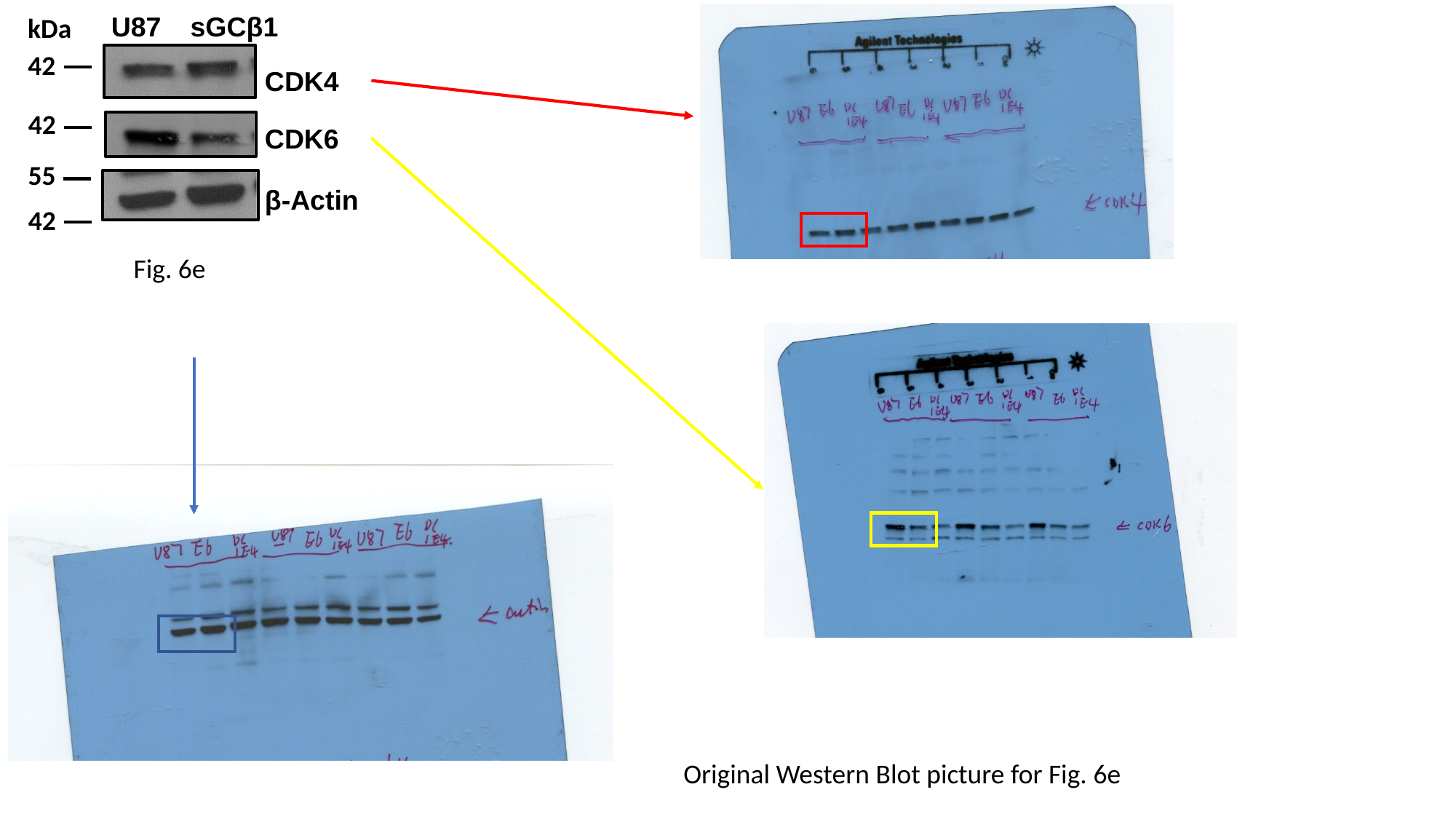

U87
sGCβ1
kDa
42
CDK4
42
CDK6
55
β-Actin
42
Fig. 6e
Original Western Blot picture for Fig. 6e

## Slide 4
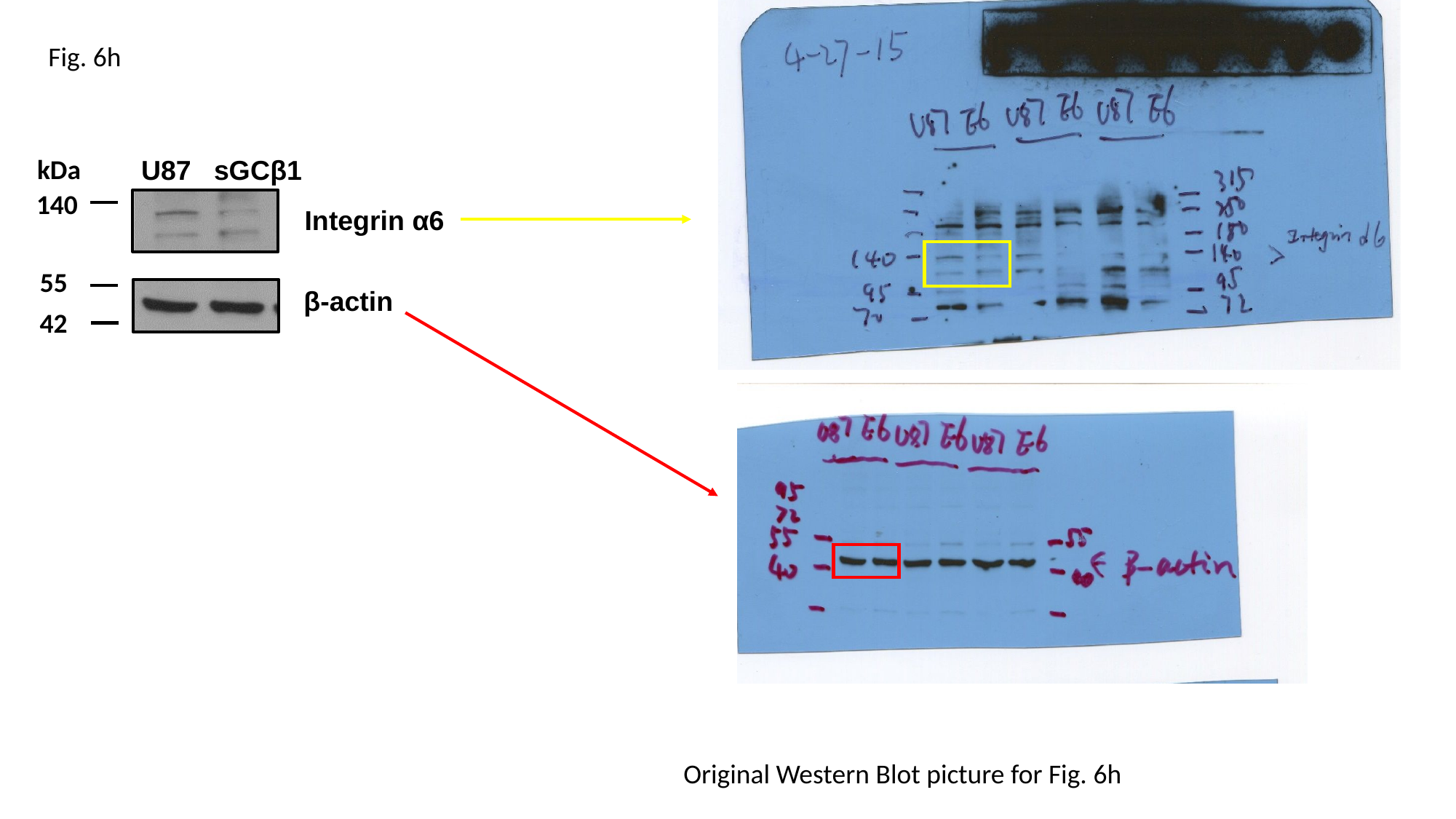

Fig. 6h
kDa
 U87 sGCβ1
140
Integrin α6
55
β-actin
42
Original Western Blot picture for Fig. 6h
