# Supplementary material for: Soluble Guanylate Cyclase β1 Subunit Represses Human Glioblastoma Growth
Source: Cancers (Basel). 2023 Mar 2;15(5):1567. doi: 10.3390/cancers15051567 (PMC10001022; doi:10.3390/cancers15051567)
Supplement: Supplementary file 1 [file cancers-15-01567-s001.zip › cancers-2104792-Supplementary Materials.pdf]

Supplementary Methods

Isolation of proteins from the cytoplasmic, soluble nuclear, and chromatin fractions

U87 cells were grown in 100 mm Petri dishes to approximately 70% confluence. The cells were trypsinized, washed twice with PBS, and resuspended in 150 μL of buffer A (10 mM HEPES, pH 7.9, 10 mM KCl, 1.5 mM $MgCl_2$, 0.34 M sucrose, 10% (v/v) glycerol, 1 mM dithiotreitol, 0.05% (v/v) NP-40, and protease and phosphatase inhibitors) for 5 min on ice.

The extract was centrifuged at 1,300×g for 5 min at 4°C. The supernatant was collected as the cytoplasm, and the pellet was washed once with 150 μL of buffer A and centrifuged. The supernatant of this centrifugation was combined with the supernatant obtained during previous centrifugation to obtain the cytoplasmic fraction with a total volume of 300 μL. Washed nuclei were used to prepare soluble nuclear and insoluble chromatin fractions.

Washed nuclei were lysed in 150 μL of buffer B (3 mM EDTA, 0.2 mM EGTA, 1 mM dithiotreitol, and protease and phosphatase inhibitors) for 10 min on ice. Soluble nuclear and chromatin fractions were separated by centrifugation at 1,700×g for 4 min at 4°C. The chromatin pellet was subsequently washed once in 150 μL of buffer B, centrifuged, and used to isolate the chromatin-binding proteins.

The chromatin fraction was resuspended in 100 μL of RIPA buffer (PBS, 1% (v/v) NP-40, 0.5% (w/v) sodium deoxycholate, 0.1% (w/v) SDS, and protease and phosphatase inhibitors), and DNA was sheared by sonication. Chromatin-binding proteins were collected by centrifugation at 12,000 rpm for 15 min at 4°C. The cytoplasmic and nuclear fractions obtained previously were also centrifuged at 12,000 rpm for 15 min at 4°C, and the pellet was discarded.

Supplementary Results

sGCβ1 is enriched in the TP53 promoter

A ChIP-seq experiment was performed as follows. A ChIP assay was performed by using sGCβ1-overexpressing cells, and the sequences of genome-wide sGCβ1-binding sites were determined. ChIP DNA was processed into a standard Illumina ChIP-seq library, which was sequenced. The 75-nt sequence reads generated by Illumina sequencing (using NextSeq 500) were mapped to the genome using the BWA algorithm with default settings. Subsequent analysis used only the reads that passed through the Illumina quality control filter, aligned with no more than 2 mismatches, and uniquely mapped to the genome. The intervals (= peaks) were determined using the MACS peak-finding algorithm. A total of 4,169 peaks were characterized in the sGCβ1 ChIP samples. The ChIP-seq data were deposited into the GEO database (accession number GSE83419). sGCβ1 was identified to be enriched in the promoter regions of many genes, including the *TP53* gene. Analysis of the distribution of sGCβ1-binding sites on the chromatin will be discussed in a subsequent publication, which is currently under preparation.

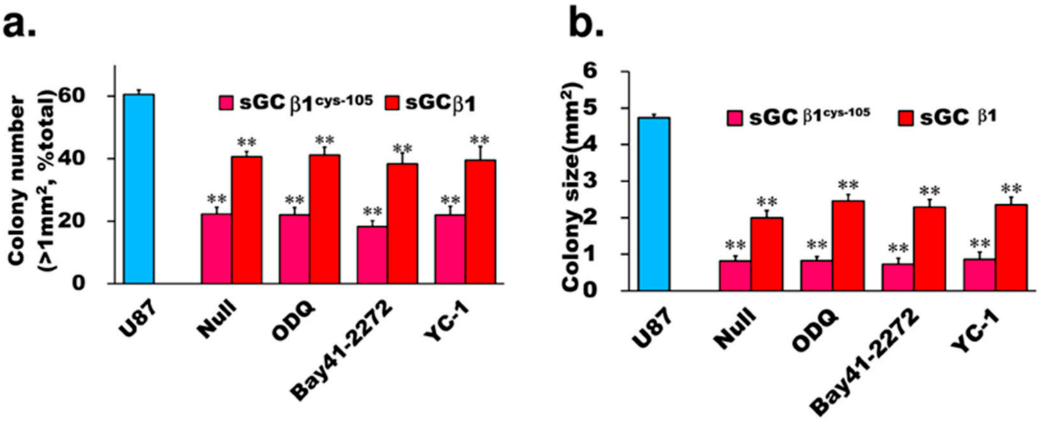

Figure S1. The sGC inhibitor ODQ (10 μM) and sGC activators Bay41-2272 (1 μM) and YC-1 (10 μM) failed to change the inhibitory effect of sGCβ1 on the number of colonies (**a**) and size of colonies of glioblastoma cells (**b**). The data are the mean ± S.E.M. *, $p < 0.05$; **, $p < 0.01$. $n = 3$-6.

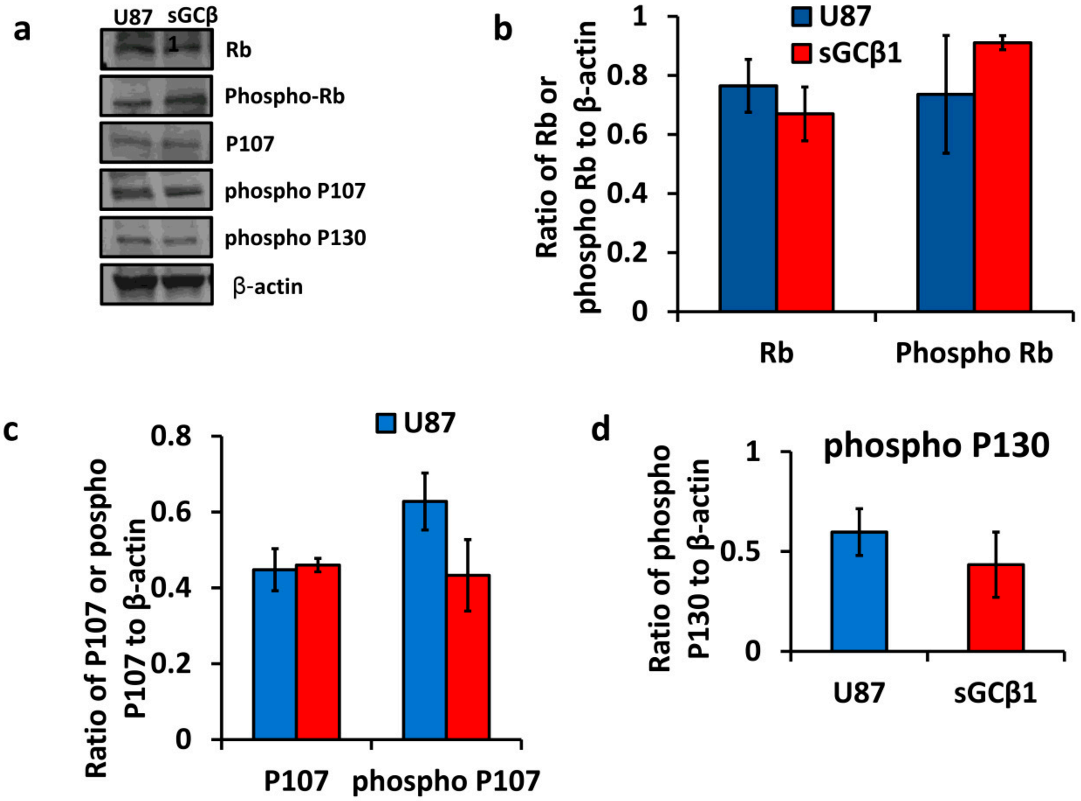

Figure S2. Immunoblotting analysis of the effect of sGCβ1 on the phosphorylation levels of p130, p107, and pRb in experimental cell preparations. sGCβ1 overexpression did not influence the phosphorylation levels of the members of the retinoblastoma family. The data are the mean ± S.E.M. $n = 3$-6.

Table S1. List of primers used in the present study.

No	Primer	Forward (F) and reverse (R)	Sequence (5'-3')
1	sGCβ1 cloning into pCDNA3.1D	F	CACCATGTACGGATTTGTGAATCAC
		R	GTCATCATCCTGCTTTGTTTCCT
2	sGCbeta1 cloning into pRS424	F	GGGGTCGACATGTACGGATTTGTGAATCAC
		R	GGGGAGCTCGTCATCATCCTGCTTTGTTTCCT

3	β-Actin cDNA	F	ATGATGATATCGCCGCGCTC
		R	CCACCATCACGCCCTGG
4	CDK4 cDNA	F	TCTGGTGACAAGTGGTGGAAC
		R	TGGTCGGCTTCAGAGTTTCC
5	CDK6 cDNA	F	AGTTCCAGAGCCTGGAGTGC
		R	ACAGCGTGACGACCACTGAG
6	ITGA6 cDNA	F	CCAACACAGGTTCTCAAGGGT
		R	CTGGCGGAGGTCAATTCTGT
7	TP53 cDNA	F	CAACAACACCAGCTCCTCTC
		R	CAAGGCCTCATTCAGCTCTC
8	TP53 promoter cloning	F	GGGGCTAGCGAGAAGAAAGGATCCAGCTG
		R	GGGAGATCTTTTCCTCCGGGAAGATGAGA
9	sGCbeta1-binding site of TP53 promoter	F	TTCTCCGCCTGCATTTCT
		R	GGGCACACCATTCAAAGAAG
10	sGCbeta1-nonbinding site of TP53 promoter	F	CCGACGCAGAGCTAAAGAAA
		R	AATTGGCGTCCGCTGTT
11	TP53 promoter deletion	F	CCTCAATCCCTCCCCTTC
		R	TCCAGAAAAAGAAATGCAGG

Table S1. Primers used for qRT-PCR.

Table S2. Antibodies used for Western blotting and immunostaining.

Antibody	Company	Catalog number
β-Actin	Santa Cruz	sc-8432
sGCβ1	Produced in-house	
sGCβ1	Sigma	G4405
sGCα1	Sigma	G4280
CDK4	Santa Cruz	sc-53636
CDK6	Santa Cruz	sc-177
Lamin A/C	Thermosfisher	MA3-1000
α-Tubulin	Sigma	T9026
p53	Santa Cruz	sc-126
Integrin α6	Santa Cruz	sc-10730
p21	Santa Cruz	sc-6246
Rb	Santa Cruz	sc-102
Phospho-Rb	Santa Cruz	sc-377540
p107	Santa Cruz	sc-250
Phospho-p107	Abcam	ab111348
Phospho-p130	Cell Signaling	4011

Table S2. Antibodies used in Western blots and immunochemical assays.

Table S3. The results of ChIP-seq analysis of the TP53 gene.

Gene	Peak length	Peak summit	Peak value	MACS p-value, -10 × log(10)
TP53	2,361	7,589,824	25	184.44

Table S3. Model-based analysis of ChIP-seq (MACS) was used to identify genome-wide locations of sGCβ1 binding. The data of ChIP-seq showed that the binding site of sGCβ1 to the *TP53* gene was enriched 1,039 bp downstream of the transcription site (MACS *p*-value 184.44).
